# Transgenically expressed *Parascaris* P-glycoprotein-11 can modulate ivermectin susceptibility in *Caenorhabditis elegans*

**DOI:** 10.1016/j.ijpddr.2015.03.003

**Published:** 2015-04-08

**Authors:** I. Jana I. Janssen, Jürgen Krücken, Janina Demeler, Georg von Samson-Himmelstjerna

**Affiliations:** Institute for Parasitology and Tropical Veterinary Medicine, Freie Universität Berlin, Robert-von-Ostertag-Str. 7-13, 14163 Berlin, Germany

**Keywords:** *Caenorhabditis elegans*, Macrocyclic lactone, Resistance, Thrashing assay, Equine parasites

## Abstract

P-glycoproteins (Pgps) are suspected to mediate drug extrusion in nematodes contributing to macrocyclic lactone resistance. This association was recently shown for *Parascaris* Pgp-11*.* Ivermectin resistance was correlated with the presence of three *pgp-11* single nucleotide polymorphisms and/or increased pgp-11 mRNA levels. In the present study, the ability of Pgp-11 to modulate ivermectin susceptibility was investigated by its expression in a *pgp-11*-deficient *Caenorhabditis elegans* strain. Expression of *Parascaris pgp-11* in two transgenic lines significantly decreased ivermectin susceptibility in a motility (thrashing) assay conducted in liquid medium. The EC_50_ values increased by 3.2- and 4.6-fold in the two lines relative to a transgenic control strain. This is the first report on the successful functional analysis of a parasitic nematode Pgp in the model organism *C. elegans*.

## Highlights

• *Parascaris* sp. pgp-11 expressed in *Caenorhabditis elegans*.
• Thrashing assay reveals increased EC_50_ for ivermectin in Pgp-11 transgenic worms.
• *C. elegans* suitable as model for functional analysis of ascarid-Pgps.

## 1 Introduction

Infections with the gastrointestinal parasite *Parascaris* sp. have a high pathogenic potential in foals and yearlings, leading occasionally to severe diseases and even death (Cribb et al., 2006). According to recent observations, the prevailing *Parascaris* species appears unclear. Therefore, the relative prevalence of *Parascaris equorum* and *Parascaris univalens* in horses is unknown (Jabbar et al., 2014; Nielsen et al., 2014). These species are morphologically indistinguishable and to date can only be differentiated by karyotyping, which was not feasible in the present study since material was collected before publication of these reports. Therefore, throughout the manuscript these parasites are referred to as *Parascaris* sp. Currently, most nematode-infected horses are treated with anthelmintics belonging to the class of macrocyclic lactones (MLs) such as ivermectin (IVM) (DiPietro et al., 1987). IVM was introduced into the market in the early 1980s and has been widely used due to its broad-spectrum activity. As a consequence, IVM resistance in *Parascaris* sp. has been reported repeatedly worldwide (von Samson-Himmelstjerna, 2012). The molecular mechanism of IVM resistance in nematodes is under active investigation with evidence suggesting polygenic mechanisms. These might be changes in gene expression levels or frequencies of alleles of genes encoding the drug target, i.e. glutamate-gated chloride channels (GluCls) (Kotze et al., 2014).

The activity of P-glycoproteins (Pgps) has also received much attention. These integral membrane proteins belong to the ABC-transporter superfamily and contain two homologous halves, each possessing six highly hydrophobic transmembrane helices. The helices constitute the transport pathway and contain several amino acid residues involved in substrate binding. The activity of these proteins is ATP-dependent. Hydrolysis of ATP drives the transport of endo- and exogenous Pgp substrates from the inner bilayer of the plasma membrane to the extracellular compartment, which leads to reduced drug concentration at the target site. Pgp substrates are usually hydrophobic or amphiphilic and have molecular weights between 330 and 4000 Da (Aller et al., 2009). Several MLs, especially IVM, interact with Pgps, as demonstrated in mammalian cell lines overexpressing murine or human Pgp genes (Lespine et al., 2007). Only recently, the expression of *Haemonchus contortus* Pgp-2 in mammalian cells was successful and its transport of MLs was described (Godoy et al., 2015).

The heterologous expression of genes encoding parasitic nematode Pgps in model nematodes such as *Caenorhabditis elegans* has not been reported yet. *C. elegans* has been widely used as a model for parasitic nematodes to screen for new drugs and to elucidate the mode of drug action (O'Reilly et al., 2014). Another option offered by the *C. elegans* system is the generation of transgenic lines for evaluating the function of genes from parasitic nematodes (Welz et al., 2011; Miltsch et al., 2012), which can be accomplished either by overexpression in the wild-type Bristol N2 strain or in strains with a specific deletion of the *C. elegans* orthologue. *C. elegans* is particularly suitable for this kind of application because it is genetically and functionally well characterised. Previous studies have reported a significantly increased IVM susceptibility of strains with a single Pgp loss-of-function mutation in a development assay (Janssen et al., 2013b), particularly for a strain lacking an intact *pgp-11*. Furthermore, its orthologue was implicated in IVM resistance in *Parascaris* since *pgp-11* was overexpressed in worms that did not respond to IVM treatment, and three non-synonymous single nucleotide polymorphisms (SNPs) within this gene correlated with decreased IVM susceptibility (Janssen et al., 2013a).

In the present study, the potential impact of Pgp-11 on IVM susceptibility in *Parascaris* sp. was assessed by the functional expression of *Parascaris* pgp-11 cDNA in a *C. elegans pgp-11* loss-of-function strain to evaluate its ability to modulate IVM resistance.

## 2 Materials and methods

### 2.1 Plasmid construction

To evaluate the impact of *Parascaris* Pgp-11 in IVM-susceptibility, a plasmid containing the complete *Parascaris* pgp-11 cDNA under control of a 3084 bp *C. elegans pgp-11* promoter fragment upstream of the *C. elegans pgp-11* start codon and the 735 bp *C. elegans* 3′-UTR of *unc-54* downstream of the cDNA sequence was constructed (Fig. S1). A control plasmid lacked the *Parascaris* pgp-11 cDNA sequence. DNA was isolated from *C. elegans* for the construction of the expression plasmids using peqGOLD TriFast™ (Peqlab, Erlangen, Germany) according to the manufacturer's recommendations. The *Parascaris* pgp–11 cDNA (accession-no.: JX308230) sequence as found in IVM susceptible worms (Janssen et al., 2013a) was amplified from a plasmid. Each region needed for plasmid construction was amplified in a PCR using gene-specific primers carrying the required restriction sites in the 5′-regions for subsequent ligation reactions. The PCRs were conducted in 50 µL reaction mixtures using 0.5 µL of Phusion Hot Start II Polymerase (Thermo Scientific), 10 µL of 5 × Phusion HF buffer, 0.5 µM of each primer, 200 µM dNTPs, and 35 ng genomic DNA (promoter and 3′-UTR) or 1 ng plasmid DNA containing the pgp-11 cDNA. An initial denaturation at 98 °C for 30 s was followed by 34 cycles of denaturation, annealing and extension according to the manufacturer's instructions. Specific annealing temperatures and extension times are provided in the supporting data (Table S1). The PCR products were cloned into the pCR4^®^-Blunt vector (Life Technologies). Plasmid DNA of the promoter and the 3′-UTR were digested with the corresponding restriction enzymes according to the manufacturer's specifications (Thermo Scientific). The linearised promoter and 3′-UTR fragments were gel-purified and sub-cloned into the expression plasmid upstream and downstream of the *Parascaris pgp-11* sequence, respectively, using T4 DNA ligase (Thermo Scientific). To generate a control plasmid lacking the *Parascaris pgp-11* sequence, this region was removed by digesting the vector with *Apa*I and *Sfi*I. The vector containing the *C. elegans* promoter and the 3′-UTR sequence was isolated by gel electrophoresis. Before re-ligation with T4 DNA ligase (Thermo Scientific), the polymerase/exonuclease activities of Phusion Hot Start II DNA polymerase (Thermo Scientific) were used to generate a blunt-end product. All plasmids were sequenced by GATC Biotech (Konstanz, Germany) to ensure that no mutations were introduced during the PCR and that the ligation sites were complete.

### 2.2 Transformation of tm0333

The mutant *C. elegans* strain deficient in *pgp-11* (tm0333) was maintained under standard conditions. Plasmids for the expression of *Parascaris* pgp-11 and the control plasmid were diluted in water and injected into the germline of young adult *C. elegans* hermaphrodites at a concentration of 50 ng/µL as described previously (Miltsch et al., 2012). A plasmid carrying a pharyngeal *gfp*-expression marker (pPD118.33, Addgene plasmid 1596: L3790) was co-injected as a transformation marker at a concentration of 25 ng/µL. Successfully transformed worms were identified by GFP fluorescence and isolated on new agar plates. Transcription of the complete sequence of *Parascaris* pgp-11 was confirmed by RT-PCR using primers and PCR conditions as described elsewhere (Janssen et al., 2013a).

### 2.3 Thrashing assay

A thrashing assay was conducted to evaluate the impact of *Parascaris* Pgp-11 for IVM-susceptibility. Young adult transgenic individuals were selected, transferred to individual wells of a 48-well plate and incubated in the dark under constant shaking (150 rpm) in S-Medium containing various IVM-concentrations (0, 1, 2.5, 5, 7.5, and 10 nM; Sigma-Aldrich, 18898; IVM B_1a_ ≥ 90%, IVM B_1b_ ≤ 5%) at 20 °C for 18 h. IVM was dissolved in dimethyl sulphoxide (DMSO) and diluted with water to a final DMSO concentration of 1%. *Escherichia coli* OP50 was available as a food source. The worms were then transferred to a well with the corresponding medium (same IVM concentration without food) and allowed to adapt to light for 1 min before movement was quantified under an inverse microscope by counting the number of body bends for 1 min. Each concentration was replicated at least three times on four separate days (n ≥ 12 for each concentration). The number of movements was normalised to the mean of the no-drug control of the same transgenic line to obtain relative motility in per cent. Regression curves were calculated using four-parameter logistic regression in GraphPad Prism 5.0 with the top and bottom values constrained to values between 0 and 100%. EC_50_ values were compared using the extra sum-of-squares *F*-test implemented in the software. *P* values < 0.05 were considered to be statistically significant.

## 3 Results and discussion

Two transgenic lines were produced by injection with the *Parascaris* pgp-11 expression plasmid (*Cel-pgp-11::Parascaris-pgp-11*(1) and *Cel-pgp-11::Parascaris-pgp-11*(2)), and another line was obtained after injection with the control plasmid (*Cel-pgp-11::control*). All lines showed semi-stable transmission of *gfp* expression, with transmission rates between 45 and 84% (Fig. S2). Transmission rates varied between lines but were apparently constant within the lines since no obvious changes in the frequency of *gfp*-positive larvae in the progeny of transgenic hermaphrodites were observed. However, exact determination of transmission rates was performed only once shortly after establishing the lines. No obvious variability of fluorescence intensity was observed between gfp-positive individuals of the same line (Fig. S2). Only individuals with *gfp* expression in the pharynx were used for further investigation. A RT-PCR targeting the full-length sequence confirmed expression of *Parascaris* pgp-11 mRNA in both transgenic lines (Fig. S3).

**Fig. 1:**
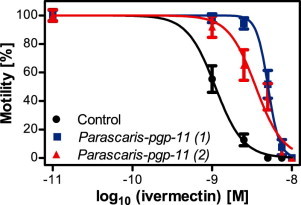
Concentration–response curves to ivermectin of the control and *Parascaris pgp-11* transgenic *Caenorhabditis elegans* in a *pgp-11*-deficient genetic background (tm0333). After incubation for 18 h in a medium containing various concentrations of ivermectin or only the vehicle DMSO (1%), the motilities of *C. elegans* worms were recorded for 1 min in liquid medium containing the same drug concentration. Transgenic lines were produced by transformation with *pgp-11* expression constructs (*Cel-pgp-11::Parascaris-pgp-11*(1) (triangle), *Cel-pgp-11::Parascaris-pgp-11*(2) (square)) or the construct lacking the *pgp-11* cDNA (*Cel-pgp-11::control* (circles)). The motility of single worms was assessed as body bends per minute. The negative control without IVM was set to 10^−11 ^M to allow log_10_ transformation of the concentrations. Values represent means ± standard error of the mean of at least 12 worms. The bottom and top values for four-parameter logistic regression were constrained to values between 0 and 100%.

A statistically significant increase (*P *<* *0.0001) in the IVM EC_50_ value was observed in the thrashing assay for both lines injected with the *Parascaris* pgp-11 expression construct, *Cel-pgp-11::Parascaris-pgp-11*(1) and *Cel-pgp-11::Parascaris-pgp-11*(2), relative to the control line *Cel-pgp-11::control* (Table 1 and Fig. 1). The EC_50_ values were increased by approximately 4.6- and 3.2-fold in the two expression constructs.

These results and data from earlier surveys strongly suggest a participation of Pgp-11 in IVM-susceptibility (Janssen et al., 2013b). The use of the *pgp-11*-deficient *C. elegans* strain tm0333 was thus appropriate in the present experiment involving the expression of the *Parascaris* orthologue to address its function in IVM susceptibility. The overall identity between the amino acid sequences of these two species is 37% (Janssen et al., 2013a). In the first and second transmembrane domains, 30% and 28% identity was observed, whereas identities of 56% and 60% were found for the first and second nucleotide-binding domain, which are in general more highly conserved. Transformation of well-known model organisms such as *C. elegans* is often the only technically feasible option for examining gene function in parasitic nematodes, since protocols for successful maintenance of transformed parasitic nematodes have not been developed yet. Accordingly, they are rarely accessible for forward or reverse genetic methods (Gilleard, 2013), mostly due to their complex life cycles that cannot be completely reproduced *in vitro*. *C. elegans* is often the expression system of choice for genes of parasitic nematodes. Functional rescues analysing anthelmintic efficacy in *C. elegans* have been conducted with ß-tubulin- and GluClα-deficient *C. elegans* strains using orthologues from *H. contortus* (Kwa et al., 1995; Glendinning et al., 2011), with *slo-1*-deficient strains containing orthologues from *Ancylostoma caninum* and *Cooperia oncophora* (Welz et al., 2011), and with an *unc-49*-deficient strain expressing the *Toxocara canis* unc-49b cDNA (Miltsch et al., 2012).

In the present study, this type of assay was successfully used to investigate the impact of *Parascaris* Pgp-11 on IVM susceptibility and decreased IVM susceptibility in a *pgp-11* loss-of-function strain of *C. elegans*. Modulation of IVM susceptibility by the injected transgene was assessed in a thrashing assay, revealing significantly increased EC_50_ values for both transgenic lines in comparison to the control line. The 3.2- and 4.6-fold increases in the EC_50_ values were similar to the 3.8-fold increase obtained in a development assay comparing the *pgp-11* loss-of-function strain with N2 wild-type *C. elegans* (Janssen et al., 2013b) but it should be stressed that changes in EC_50_ values cannot be directly compared between different types of assays. As within each generation a fraction of the transgenic individuals loses the transgene, it is difficult to conduct a development assay with lines carrying extra-chromosomal transgenes. For that reason, a thrashing assay was performed which uses individual worms identified as transgenic due to *gfp*-expression and not populations of ca. 100 worms as statistical unit. In comparison to body-bend assays conducted on agar plates, worms move more rapidly in thrashing assays (Miller et al., 1996) resulting in a broader dynamic range of the assay. In addition, a liquid medium as it was used here, probably allows a more reproducible and homogenous IVM-distribution than an agar-based medium.

ABC transporters are known for their multi-drug-resistance activity in eukaryotes and prokaryotes. They are able to mediate transport in both directions in prokaryotic cells, but only act as exporters from the cytosolic compartment in eukaryotes (Davidson et al., 2008). Several nematode Pgps have been suspected to be involved in resistance to IVM. Apart from one functional study using a recombinant Pgp (Godoy et al., 2015), most of the records are descriptive and report changes in expression levels or frequency of alleles. Only a few Pgps appear to be of particular importance for IVM detoxification and the development of resistance. For example, increased expression in ML-resistant isolates has been reported for *pgp-9* in *Teladorsagia circumcincta* and *H. contortus* and for *pgp-2* in *H. contortus* (Dicker et al., 2011; Williamson et al., 2011). Furthermore, *pgp-11* was expressed at higher levels in an IVM-resistant isolate of *C. oncophora* (De Graef et al., 2013). Comparable results were obtained for *pgp-1* of *Onchocerca volvulus* (Huang and Prichard, 1999), which is in fact an orthologue of *C. elegans pgp-11* (Ardelli and Prichard, 2013). Very recently, Godoy et al. (2015) and Kaschny et al. (2015) have shown that recombinant *H. contortus* Pgp-2 and *Cylicocyclus elongatus* Pgp-9 interact directly with MLs and that the intensity of interaction depends on the particular ML.

The decrease in susceptibility observed in the transgenic *C. elegans* model system clearly demonstrates that *Parascaris* Pgp-11 can contribute to the response to treatment with IVM. Nevertheless, this increase on its own, even if it occurs at a similar level in *Parascaris* sp., might not be high enough to entirely account for the phenotypically apparent IVM resistance levels observed in *Parascaris* populations in the field or in trichostrongyloid species of sheep (Demeler et al., 2013). The combined effects of changes in several paralogues, however, may produce higher resistance levels. Considering the genetic background encoding 13 additional, functional Pgps, and assuming that different Pgps have overlapping substrate spectra, an increase in the EC_50_ value of more than 4.6-fold probably cannot be expected.

In the future, the three SNPs within *Parascaris pgp-11* that have been correlated with an IVM resistance phenotype (Janssen et al., 2013a) should be analysed regarding their individual effects on IVM susceptibility. For this approach, the MosSCI recombination system is an efficient tool to insert transgenes into defined chromosomal locations. This method is suitable to eliminate confounding effects of transgene transmission to the next generation, copy number, and integration-site dependant differences in expression levels (Frokjaer-Jensen et al., 2008).

To our knowledge this is the first report on the successful functional analysis of a parasitic nematode Pgp in the model organism *C. elegans*. The results described in this study provide an important insight into the impact of a single Pgp from a parasitic nematode in the mechanism of IVM detoxification. The current *C. elegans* expression system still has relevant limitations, but it allows the functional analysis of genes associated with anthelmintic resistance in a model organism resembling parasitic nematodes as closely as currently possible.

## Conflict of interest

The authors declared that there is no conflict of interest.

## Figures and Tables

**Table 1:** Effect of *Parascaris pgp-11* expression on ivermectin susceptibility in *Caenorhabditis elegans pgp-11* deficient background (strain tm0333) recorded by measurement of individual motility (body bends).

Line	*Cel-pgp-11::control*	*Cel-pgp-11::Parascaris-pgp-11*(1)	*Cel-pgp-11::Parascaris-pgp-11*(2)
EC_50_^a^ [µM] (95% CI^b^)	1.095 (0.96–1.24)	5.033 (4.71–5.38)	3.523 (2.97–4.18)
*R*^2^	0.7469	0.7978	0.676
*P*	–	<0.0001	<0.0001
Fold change of EC_50_ value from control line	–	4.6	3.2

a EC_50_, 50% effective concentration.

b CI, confidence interval.

## Appendix Supplementary material

Fig. S1 Scheme of restriction sites (*Not*I, *Apa*I, *Sfi*I and *Sbf*I) within the pCR™4-TOPO^®^TA vector after insertion of the sequences for *Parascaris pgp-11*, *Caenorhabditis elegans pgp-11* promoter and *C. elegans* 3′-UTR of *unc-54*.

Fig. S2 Fluorescence photographs of two transgenic lines on agar plates. A line with a low (A) and a high (B) transmission rate is shown. In (B) different live cycle stages can be observed.

Fig. S3 Gel electrophoresis showing *Parascaris* pgp-11 mRNA expression in *Caenorhabditis elegans* after RT-PCR. For RNA isolation of each *C. elegans* line, approximately 50 GFP-positive worms were selected individually and homogenised with a speed mill (Analytik Jena). The RNA extraction was conducted using the NucleoSpin RNA XS kit (Macherey and Nagel). Lane M: DNA 1 kb Marker, lane 1: *Parascaris* pgp-11 expressed in line *Cel-pgp-11::Parascaris-pgp-11*(1), lane 2: no RT-PCR control (NRT) of *Cel-pgp-11::Parascaris-pgp-11*(1), lane 3: *Parascaris* pgp-11 expression in line *Cel-pgp-11::Parascaris-pgp-11*(2), lane 4: NRT of *Cel-pgp-11::Parascaris-pgp-11*(2), lane 5: *Parascaris* pgp-11 expression in *Cel-pgp-11::control*, lane 6: NRT of *Cel-pgp-11::control*, lane 7: no template control.

Table S1 Primer sequences used for the amplification of the *C. elegans pgp-11* promoter region, *Parascaris*Pgp-11, and the *C. elegans* 3′-UTR of *unc-54* and the corresponding PCR conditions.

## References

[bib0010] Aller S.G., Yu J., Ward A., Weng Y., Chittaboina S., Zhuo R. (2009). Structure of P-glycoprotein reveals a molecular basis for poly-specific drug binding. Science.

[bib0015] Ardelli B.F., Prichard R.K. (2013). Inhibition of P-glycoprotein enhances sensitivity of *Caenorhabditis elegans* to ivermectin. Vet. Parasitol.

[bib0020] Cribb N.C., Coté N.M., Bouré L.P., Peregrine A.S. (2006). Acute small intestinal obstruction associated with *Parascaris equorum* infection in young horses: 25 cases (1985–2004). N. Z. Vet. J..

[bib0025] Davidson A.L., Dassa E., Orelle C., Chen J. (2008). Structure, function, and evolution of bacterial ATP-binding cassette systems. Microbiol. Mol. Biol. Rev.

[bib0030] De Graef J., Demeler J., Skuce P., Mitreva M., von Samson-Himmelstjerna G., Vercruysse J. (2013). Gene expression analysis of ABC transporters in a resistant *Cooperia oncophora* isolate following *in vivo* and *in vitro* exposure to macrocyclic lactones. Parasitology.

[bib0035] Demeler J., Gill J.H., von Samson-Himmelstjerna G., Sangster N.C. (2013). The in vitro assay profile of macrocyclic lactone resistance in three species of sheep trichostrongyles. Int. J. Parasitol. Drugs Drug Resist.

[bib0040] Dicker A.J., Nisbet A.J., Skuce P.J. (2011). Gene expression changes in a P-glycoprotein (*Tci-pgp-9*) putatively associated with ivermectin resistance in *Teladorsagia circumcincta*. Int. J. Parasitol.

[bib0045] DiPietro J.A., Lock T.F., Todd K.S., Reuter V.E. (1987). Evaluation of ivermectin paste in the treatment of ponies for *Parascaris equorum* infections. J. Am. Vet. Med. Assoc.

[bib0050] Frokjaer-Jensen C., Davis M.W., Hopkins C.E., Newman B.J., Thummel J.M., Olesen S.P. (2008). Single-copy insertion of transgenes in Caenorhabditis elegans. Nat. Genet.

[bib0055] Gilleard J.S. (2013). *Haemonchus contortus* as a paradigm and model to study anthelmintic drug resistance. Parasitology.

[bib0060] Glendinning S.K., Buckingham S.D., Sattelle D.B., Wonnacott S., Wolstenholme A.J. (2011). Glutamate-gated chloride channels of *Haemonchus contortus* restore drug sensitivity to ivermectin resistant *Caenorhabditis elegans*. PLoS ONE.

[bib0065] Godoy P., Lian J., Beech R.N., Prichard R.K. (2015). Haemonchus contortus P-glycoprotein-2: in situ localisation and characterisation of macrocyclic lactone transport. Int. J. Parasitol.

[bib0070] Huang Y.J., Prichard R.K. (1999). Identification and stage-specific expression of two putative P-glycoprotein coding genes in *Onchocerca volvulus*. Mol. Biochem. Parasitol.

[bib0075] Jabbar A., Littlewood D.T., Mohandas N., Briscoe A.G., Foster P.G., Muller F. (2014). The mitochondrial genome of *Parascaris univalens –* implications for a “forgotten” parasite. Parasit. Vectors.

[bib0080] Janssen I.J.I., Krücken J., Demeler J., Basiaga M., Kornas S., von Samson-Himmelstjerna G. (2013). Genetic variants and increased expression of *Parascaris equorum* P-glycoprotein-11 in populations with decreased ivermectin susceptibility. PLoS ONE.

[bib0085] Janssen I.J.I., Krücken J., Demeler J., von Samson-Himmelstjerna G. (2013). *Caenorhabditis elegans*: modest increase of susceptibility to ivermectin in individual P-glycoprotein loss-of-function strains. Exp. Parasitol.

[bib0090] Kaschny M., Demeler J., Janssen I.J.I., Kuzmina T.A., Besognet B., Kanellos T. (2015). Macrocyclic lactones differ in interaction with recombinant P-glycoprotein 9 of the parasitic nematode *Cylicocyclus elongatus* and ketokonazole in a yeast growth assay. PLoS Pathog.

[bib0095] Kotze A.C., Hunt P.W., Skuce P., von Samson-Himmelstjerna G., Martin R.J., Sager H. (2014). Recent advances in candidate-gene and whole-genome approaches to the discovery of anthelmintic resistance markers and the description of drug/receptor interactions. Int. J. Parasitol. Drugs Drug Resist.

[bib0100] Kwa M.S.G., Veenstra J.G., Van Dijk M., Roos M.H. (1995). Beta-tubulin genes from the parasitic nematode *Haemonchus contortus* modulate drug resistance in *Caenorhabditis elegans*. J. Mol. Biol.

[bib0105] Lespine A., Martin S., Dupuy J., Roulet A., Pineau T., Orlowski S. (2007). Interaction of macrocyclic lactones with P-glycoprotein: structure-affinity relationship. Eur. J. Pharm. Sci.

[bib0110] Miller K.G., Alfonso A., Nguyen M., Crowell J.A., Johnson C.D., Rand J.B. (1996). A genetic selection for *Caenorhabditis elegans* synaptic transmission mutants. Proc. Natl. Acad. Sci. U.S.A..

[bib0115] Miltsch S.M., Krücken J., Demeler J., Janssen I.J.I., Krüger N., Harder A. (2012). Decreased emodepside sensitivity in *unc-49* gamma-aminobutyric acid (GABA)-receptor-deficient *Caenorhabditis elegans*. Int. J. Parasitol.

[bib0120] Nielsen M.K., Wang J., Davis R., Bellaw J.L., Lyons E.T., Lear T.L. (2014). Parascaris univalens – a victim of large-scale misidentification?. Parasitol. Res.

[bib0125] O'Reilly L.P., Luke C.J., Perlmutter D.H., Silverman G.A., Pak S.C. (2014). C. elegans in high-throughput drug discovery. Adv. Drug Deliv. Rev.

[bib0130] von Samson-Himmelstjerna G. (2012). Anthelmintic resistance in equine parasites – detection, potential clinical relevance and implications for control. Vet. Parasitol.

[bib0135] Welz C., Krüger N., Schniederjans M., Miltsch S.M., Krücken J., Guest M. (2011). SLO-1-channels of parasitic nematodes reconstitute locomotor behaviour and emodepside sensitivity in *Caenorhabditis elegans slo-1* loss of function mutants. PLoS Pathog.

[bib0140] Williamson S.M., Storey B., Howell S., Harper K.M., Kaplan R.M., Wolstenholme A.J. (2011). Candidate anthelmintic resistance-associated gene expression and sequence polymorphisms in a triple-resistant field isolate of *Haemonchus contortus*. Mol. Biochem. Parasitol.

