# Supplementary material for: Transgenically expressed Parascaris P-glycoprotein-11 can modulate ivermectin susceptibility in Caenorhabditis elegans
Source: Int J Parasitol Drugs Drug Resist. 2015 Apr 8;5(2):44–7. doi: 10.1016/j.ijpddr.2015.03.003 (PMC4401813; doi:10.1016/j.ijpddr.2015.03.003)
Supplement: Fig. S1 — Scheme of restriction sites (NotI, ApaI, SfiI and SbfI) within the pCR™4-TOPO®TA vector after insertion of the sequences for Parascaris pgp-11, Caenorhabditis elegans pgp-11 promoter and C. elegans 3′-UTR of unc-54. [file mmc1.pdf]

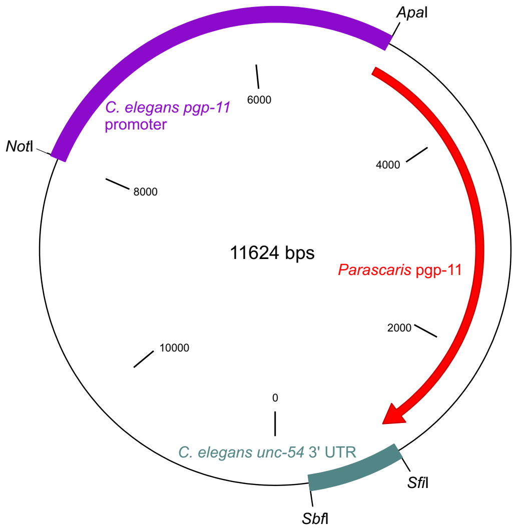

Fig. S1. Scheme of restriction sites (*Not*I, *Apa*I, *Sfi*I and *Sbf*I) within the pCR™4-TOPO®TA vector after insertion of the sequences for *Parascaris pgp-11*, *Caenorhabditis elegans pgp-11* promoter and *C. elegans* 3'-UTR of *unc-54*.
