# Supplementary material for: Transgenically expressed Parascaris P-glycoprotein-11 can modulate ivermectin susceptibility in Caenorhabditis elegans
Source: Int J Parasitol Drugs Drug Resist. 2015 Apr 8;5(2):44–7. doi: 10.1016/j.ijpddr.2015.03.003 (PMC4401813; doi:10.1016/j.ijpddr.2015.03.003)
Supplement: Fig. S2 — Fluorescence photographs of two transgenic lines on agar plates. A line with a low (A) and a high (B) transmission rate is shown. In (B) different live cycle stages can be observed. [file mmc2.pdf]

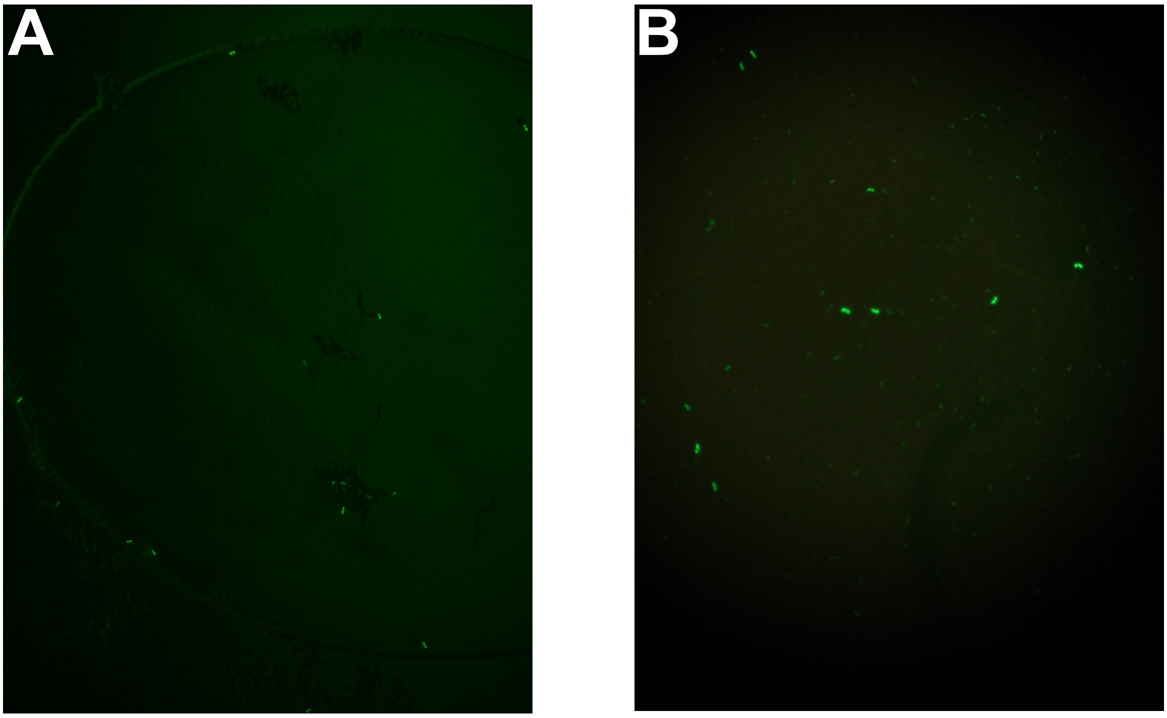

Fig. S2. Fluorescence photographs of two transgenic lines on agar plates. A line with a low (A) and a high (B) transmission rate are shown. In (B) different live cycle stages can be observed.
