# Supplementary material for: Transgenically expressed Parascaris P-glycoprotein-11 can modulate ivermectin susceptibility in Caenorhabditis elegans
Source: Int J Parasitol Drugs Drug Resist. 2015 Apr 8;5(2):44–7. doi: 10.1016/j.ijpddr.2015.03.003 (PMC4401813; doi:10.1016/j.ijpddr.2015.03.003)
Supplement: Fig. S3 — Gel electrophoresis showing Parascaris pgp-11 mRNA expression in Caenorhabditis elegans after RT-PCR. For RNA isolation of each C. elegans line, approximately 50 GFP-positive worms were selected individually and homogenised with a speed mill (Analytik Jena). The RNA extraction was conducted using the NucleoSpin RNA XS kit (Macherey and Nagel). Lane M: DNA 1 kb Marker, lane 1: Parascaris pgp-11 expressed in line Cel-pgp-11::Parascaris-pgp-11(1), lane 2: no RT-PCR control (NRT) of Cel-pgp-11::Parascaris-pgp-11(1), lane 3: Parascaris pgp-11 expression in line Cel-pgp-11::Parascaris-pgp-11(2), lane 4: NRT of Cel-pgp-11::Parascaris-pgp-11(2), lane 5: Parascaris pgp-11 expression in Cel-pgp-11::control, lane 6: NRT of Cel-pgp-11::control, lane 7: no template control. [file mmc3.pdf]

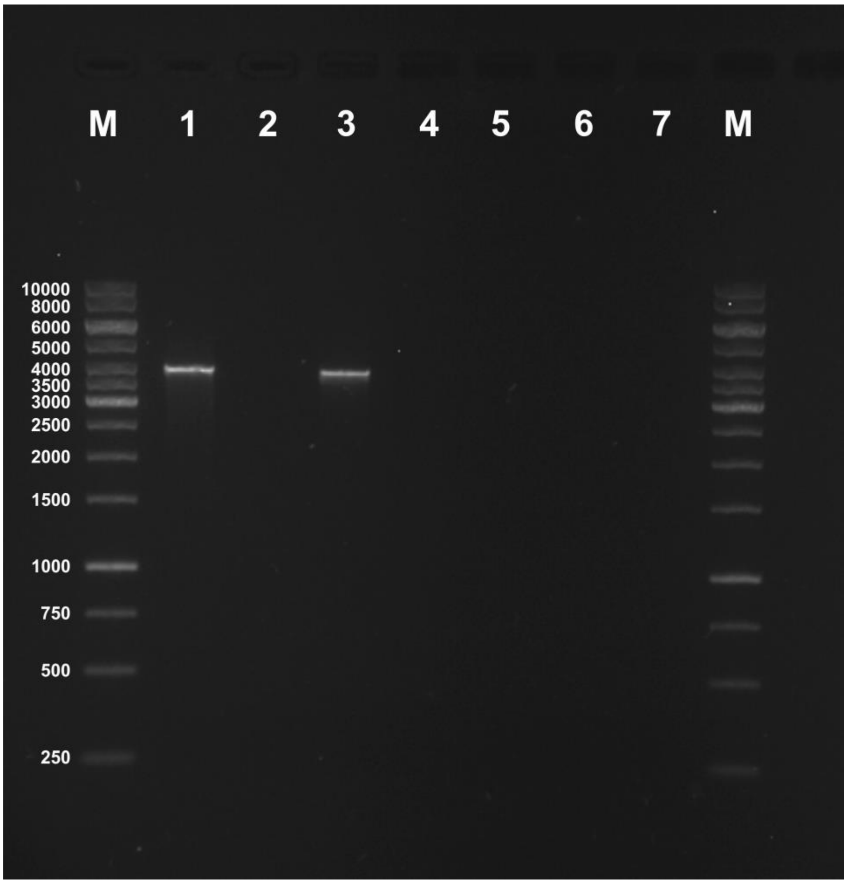

Fig. S3. Gel electrophoresis showing *Parascaris* pgp-11 mRNA expression in *Caenorhabditis elegans* after RT-PCR. For RNA isolation of each *C. elegans* line, approximately 50 GFP-positive worms were selected individually and homogenised with a speed mill (Analytik Jena). The RNA extraction was conducted using the NucleoSpin RNA XS kit (Macherey and Nagel). Lane M: DNA 1 kb Marker, lane 1: *Parascaris* pgp-11 expressed in line *Cel-pgp-11::Parascaris-pgp-11*(1), lane 2: no RT-PCR control (NRT) of *Cel-pgp-11::Parascaris-pgp-11*(1), lane 3: *Parascaris* pgp-11 expression in line *Cel-pgp-11::Parascaris-pgp-11*(2), lane 4: NRT of *Cel-pgp-11::Parascaris-pgp-11*(2), lane 5 *Parascaris* pgp-11 expression in *Cel-pgp-11::control*, lane 6 NRT of *Cel-pgp-11::control*, lane 7 no template control.
