# Supplementary material for: Transgenically expressed Parascaris P-glycoprotein-11 can modulate ivermectin susceptibility in Caenorhabditis elegans
Source: Int J Parasitol Drugs Drug Resist. 2015 Apr 8;5(2):44–7. doi: 10.1016/j.ijpddr.2015.03.003 (PMC4401813; doi:10.1016/j.ijpddr.2015.03.003)
Supplement: Table S1 — Primer sequences used for the amplification of the C. elegans pgp-11 promoter region, ParascarisPgp-11, and the C. elegans 3′-UTR of unc-54 and the corresponding PCR conditions. [file mmc4.pdf]

Supplemental material

Table S1

Primer sequences used for the amplification of the *C. elegans pgp-11* promoter region, *Parascaris*Pgp-11, and the *C. elegans* 3'-UTR of *unc-54* and the corresponding PCR conditions

Sequence/Gene	Acc. No[a]	Primer (Name and Sequence 5'-3')	Size (bp)[b]	AT[c]	ET[d]
Cosmid DH11	Z49126	*Cel*PromPgp-11-NotI-Se<br>GCGGCCGCAGTCAGGTGTCCTCTTTATTGAGGG	3084	68	90
		*Cel*PromPgp-11-ApaI-AS<br>GGGCCCGTTCTCTTAAATACACACTGAATTTGAG			
Pgp-11	JX308230	*Parascaris*Pgp-11-ApaI-Se<br>GGGCCCAAAATGGAACTGGAAGCAAAACAACCAGTTGG	3858	75	60
		*Parascaris*Pgp-11-SfiI-AS<br>GGCCTTTTTGGCCTCACGACGTGAGGTCCTGCTTTCGG			
UTR	J01050	*Cel*3UTR-SfiI-Se<br>GGCCAAAAAGGCCCATCTCGCGCCCGTGCCTCT	735	67	30
		*Cel*3UTR-SbfI-AS<br>CCTGCAGGAAACAGTTATGTTTGGTATATTGGGAAT			

[a] Accession number
[b] Size of the amplified PCR products in bp
[c] Annealing temperature in °C
[d] Elongation time (ET) in seconds
